# Plasma mannose as a novel marker of myocardial infarction across different glycaemic states: a case control study

**DOI:** 10.1186/s12933-022-01630-5

**Published:** 2022-09-23

**Authors:** Elena Fortin, Giulia Ferrannini, Beatrice Campi, Linda Mellbin, Anna Norhammar, Per Näsman, Alessandro Saba, Ele Ferrannini, Lars Rydén

**Affiliations:** 1 Division of Cardiology, Department of Medicine K2, Karolinska Institutet, Norrbacka S1:02, 171 76, Stockholm, Sweden; 2 National Research Council Institute of Clinical Physiology, Pisa, Italy; 3 Heart Vascular and Neuro Theme, Karolinska University Hospital, Stockholm, Sweden; 4 Capio St Görans Hospital, Stockholm, Sweden; 5 Center for Safety Research, KTH Royal Institute of Technology, Stockholm, Sweden; 6 Mass Spectrometry Laboratory, Department of Pathology, University of Pisa, Pisa, Italy; 7 Clinical Pathology Laboratory, Santa Chiara University Hospital, Pisa, Italy

**Keywords:** Glucose perturbations, Mannose, Myocardial infarction, Risk marker

## Abstract

**Background:**

Plasma mannose, an emerging novel biomarker of insulin resistance, is associated with both diabetes mellitus and coronary atherosclerosis, but the relationship between mannose concentrations and myocardial infarction (MI) across different glycaemic states remains to be elucidated. The aim of this study was to investigate the independent association between mannose and a first MI in a group of subjects characterized according to their glycaemic state.

**Methods:**

Fasting plasma mannose concentrations were analysed in 777 patients 6–10 weeks after a first myocardial infarction and in 770 matched controls by means of high-performance liquid chromatography coupled to tandem mass spectrometry. Participants without known diabetes mellitus were categorized by an oral glucose tolerance test (OGTT) as having normal glucose tolerance (NGT, n = 1045), impaired glucose tolerance (IGT, n = 246) or newly detected type 2 diabetes (T2DM, n = 112). The association between mannose and MI was investigated across these glycaemic states by logistic regression.

**Results:**

Mannose levels increased across the glycaemic states (p < 0.0001) and were significantly associated with a first MI in the whole study population (odds ratio, OR: 2.2; 95% CI 1.4 to − 3.5). Considering the different subgroups separately, the association persisted only in subjects with NGT (adjusted OR: 2.0; 95% CI 1.2–3.6), but not in subgroups with glucose perturbations (adjusted OR: 1.8, 95% CI 0.8–3.7).

**Conclusions:**

Mannose concentrations increased across worsening levels of glucose perturbations but were independently associated with a first MI only in NGT individuals. Thus, mannose might be a novel, independent risk marker for MI, possibly targeted for the early management of previously unidentified patients at high cardiovascular risk.

**Supplementary Information:**

The online version contains supplementary material available at 10.1186/s12933-022-01630-5.

## Background

With almost 18 million deaths per year, which corresponds to one third of the total global mortality estimated for 2021, cardiovascular disease (CVD) is currently the biggest killer worldwide [1], representing an important challenge for contemporary healthcare systems. Diabetes mellitus (DM), a chronic disease with a prevalence expected to climb from presently 537 million people to 783 million in 2045 [2] is a major cause of morbidity and mortality due to micro- and macrovascular complications [3, 4]. The coronary risk is present before the onset of DM and increased already in people with impaired glucose tolerance (IGT) [5, 6]. Therefore, it is alarming that abnormalities in glucose metabolism frequently remain undetected, delaying the initiation of potentially life-saving preventive measures such as lifestyle changes and pharmacological interventions [7, 8]. In this setting, and in line with the contemporary concept of “personalized medicine”, research on novel biomarkers that may improve cardiovascular (CV) risk prediction pursues the goal of prompt interventions and a more effective management of high-risk individuals.

Elevated mannose, a C2 epimer of glucose, has been associated with incident type 2 diabetes (T2DM) and CVD [9], and has recently emerged as a novel, insulin-regulated, biomarker of insulin resistance [10]. Hence, this metabolite may be a new marker able to early identify insulin-resistant people at high CV risk [10, 11]. That mannose is related to incident CVD and glucose perturbations is reinforced by a recent validation study comprising patients with prevalent coronary artery disease (CAD), where plasma mannose was correlated not only with CAD severity but also with adverse CV outcomes and mortality [12].

The relationship between mannose and CAD has, however, not been studied in relation to the glycaemic state. Based on the emerging notion that insulin resistance and hyperinsulinemia per se may predict CVD independent of hyperglycemia [13] and that mannose is a valuable marker of insulin resistance [10], high mannose may increase the risk of a first myocardial infarction (MI) not only in patients with diabetes, but also in those with some degrees of insulin resistance but without any manifest signs of glucose intolerance. To validate this hypothesis, in the present investigation we explored the associations between mannose levels and a first MI in people with different degrees of glucose perturbations.

## Methods

### Study population

The Periodontitis and Its Relation to Coronary Artery Disease (PAROKRANK) Study is a Swedish multicenter case–control investigation described in detail elsewhere [14]. In short, 805 patients < 75 years of age admitted to 17 Swedish hospitals with a diagnosis of a first MI based on international criteria [15] were recruited between May 2010 and February 2014. Exclusion criteria were prior MI, prior heart valve replacement and any other condition that, according to the judgement of the investigator, could limit the ability to cope with the protocol. During the same period, 805 sex- age- (± 3 months) and area-matched controls, randomly selected from the national population registry, were investigated shortly after the outpatient visit of their corresponding patient. The controls had to be free from a history of prior MI and heart valve replacement. Medical information was obtained from the hospital records for the patients and by standardized interviews of the controls. Study visits and laboratory investigations were carried out 6–10 weeks after the MI for patients, and for the matched controls within 10 days after the outpatient visit of their corresponding patient.

**Fig. 1:**
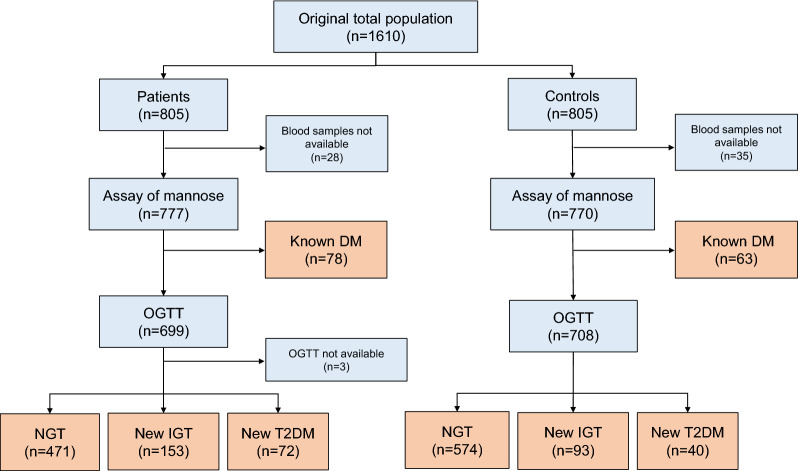
Flowchart showing how study participants were categorized following the oral glucose tolerance test. DM, Diabetes mellitus; OGTT, Oral glucose tolerance test; NGT, Normal glucose tolerance; IGT, Impaired glucose tolerance; T2DM, type 2 diabetes mellitus

All study participants without known DM underwent an oral glucose tolerance test (OGTT) and were categorized as having normal glucose tolerance (NGT), IGT or T2DM according to World Health Organization (WHO) classification, thus based on post-load glucose but not on glycated haemoglobin A1c (HbA1c)-criteria [16, 17]. Following the OGTT results, four groups, i.e., normal glucose tolerance (NGT), IGT, new T2DM and known DM were considered in the present study (see Fig. 1). Study participants with either new IGT, new T2DM or known DM were referred to as having “glucose perturbations”. Subjects with known DM had either type 1 or type 2 DM.

### Laboratory analyses

Blood samples were collected during the outpatient visit after fasting and smoking abstinence for at least 12 h. Biochemical parameters, including complete blood count, plasma(P)-lipids (total and high-density lipoprotein cholesterol and triglycerides), P-creatinine, P-fibrinogen, P-glucose, and HbA1c were analyzed at the local laboratories of each hospital. In the OGTT, P-glucose was assayed in the fasting state (fasting plasma glucose—FPG), and after 30 and 120 min (Two hours post-load glucose—2 h-PG) following ingestion of 75 g of glucose in 200 mL water and using a bedside point-of-care system (HemoCue 201 System; HemoCue AB, Ängelholm, Sweden). High-sensitivity C-reactive protein (hs-CRP) was assayed at a central laboratory (Redhot Diagnostics, Södertälje, Sweden) by an ELISA method (MP Biomedicals, New York, USA). Plasma (6 mL) and whole blood (4 mL) were also collected and stored in a central biobank at Karolinska Institutet at − 70 °C. Mannose was assayed by high-performance liquid chromatography coupled to tandem mass spectrometry (HPLC–MS-MS) at the Mass Spectrometry laboratory in Pisa, Italy. In brief, plasma mannose was separated from its epimers, glucose and galactose, by HPLC and LC–MS-MS was then used to quantify its concentrations [18].

### Statistical analysis

Continuous variables are presented as median and interquartile range; categorical data are given as number and percentages. Differences in baseline continuous data between patients and controls were compared using Mann–Whitney U test; proportions were compared using a χ^2^ test. The Mann–Whitney U test was used to carry out comparisons of mannose levels between patients and controls, both according to and regardless of their glycaemic status, and between individuals with different glycaemic states in the whole population. The difference in mannose concentrations across the four glucose tolerance groups (combining patients and controls) was determined by the Kruskal–Wallis test. Statistical significance was considered for a two-sided p < 0.05. The relationship between logarithmically transformed mannose concentrations and glycaemic parameters (i.e., HbA1c, FPG, 2hPG), body mass index (BMI), waist circumference, fibrinogen and hs-CRP were assessed by Spearman’s correlation coefficient. The association between log-transformed mannose concentrations and a first MI (patients vs controls) was investigated by a conditional logistic regression model, adjusting for covariates selected by using backward elimination method with an alpha level of 0.1 (i.e., smoking status, family history of cardiovascular disease and education level). The same association was assessed within each of the four glucose tolerance groups as well as in all patients with glucose perturbations (IGT, newly detected DM and known DM together), adjusting for sex, age, smoking status, family history of cardiovascular disease and education level. Different regression models were fitted to try to account for all sensible confounders while avoiding overadjustments. The OR and corresponding 95% CIs to estimate the association between mannose levels and a first MI in the total cohort was also calculated for a one-SD increase in mannose levels by use of logistic regression. The optimal cut-off of mannose concentrations was obtained by the maximum Youden’s J statistic on the adjusted receiver operator characteristics (ROC) curve testing the diagnostic performance in diagnosing a first MI, including a matching concordance index in the model [19]. All analyses were performed using STATA version IC/16.1.

## Results

### Clinical characteristics

Among the original 1610 study participants (805 patients and 805 controls), samples for mannose assays were available in 1547 subjects (777 patients and 770 controls), who represent the current study population. Following the OGTT, patients and controls without known DM were classified according to their glycaemic state as described in the flowchart in Fig. 1.

Newly detected glucose perturbations (i.e., new IGT or new T2DM) were significantly more common in patients than in controls (29.0% vs. 17.3%): 19.7% patients and 12.1% controls had new IGT (p < 0.0001), and 9.3% patients and 5.2% controls had new T2DM (p = 0.0018).

Pertinent baseline clinical characteristics of the total population (patients vs controls) are presented in Table 1.

**Table 1:** Pertinent baseline characteristics of the study participants

Variables	Patients (n = 777)	Controls (n = 770)	P-value	Missing
Age, years	63 (58–67)	64 (58–68)	0.54	0
Male sex, n (%)	630 (81.1)	623 (80.8)	0.89	0
Marital status				
Single	118 (15.2)	77 (10.0)		
Married	574 (74.0)	610 (79.4)	0.008	2
Divorced/widowed	84 (10.8)	82 (10.6)		
Education level				
1–12 years	511 (66.0)	471 (61.3)	0.055	6
University	263 (34.0)	297 (38.7)		
Medical history^a^				
Known family history of CVD^b^	293 (43.6)	178 (26.6)	< 0.0001	207
Hypertension	274 (35.4)	253 (33.0)	0.32	7
Diabetes mellitus	78 (10.1)	63 (8.2)	0.20	5
Peripheral artery disease	19 (2.4)	10 (1.3)	0.096	0
Stroke	22 (2.8)	17 (2.2)	0.43	6
Rheumatic disease	156 (20.5)	132 (17.3)	0.11	21
Pulmonary disease	106 (14.0)	85 (11.1)	0.09	25
Kidney disease	30 (3.9)	30 (3.9)	0.98	0
Cancer	62 (8.0)	57 (7.4)	0.67	0
Smoking habits (patients at admission)				
Current	200 (26.2)	91 (11.8)	< 0.0001	15
Previous (> 1 month)	273 (35.8)	345 (44.7)		
Never	289 (37.9)	335 (43.5)		
Waist circumference, cm	99 (92–106)	98 (91–106)	0.14	3
Body Mass Index, kg/m^2^	26.6 (24.5–29.3)	26.5 (24.1–29.1)	0.30	0
Blood pressure, mmHg				
Systolic	130 (120–140)	136 (125–148)	< 0.0001	1
Diastolic	78 (70–110)	83.0 (77–90)	< 0.0001	1
Laboratory values				
Cholesterol, mmol/L	3.8 (3.3–4.4)	5.5 (4.8–6.2)	< 0.0001	9
Triglycerides, mmol/L	1.1 (0.9–1.6)	1.2 (0.9–1.6)	< 0.0001	11
HDL-cholesterol, mmol/L	1.2 (1.0–1.4)	1.4 (1.2–1.7)	< 0.0001	10
Haemoglobin, g/L	142 (134–150)	146 (138–154)	< 0.0001	13
FPG, mmol/L	5.8 (5.3–6.4)	5.4 (4.9–5.9)	< 0.0001	25
2 h-PG, mmol/L^c^	6.6 (5.3–8.1)	5.8 (4.7–7.1)	< 0.0001	179
HbA1c, mmol/mol	39 (37–43)	38 (35–41)	< 0.0001	25
HbA1c, %	5.7 (5.5–6.1)	5.6 (5.4–5.9)	< 0.0001	25
Mannose, μmol/L	74.5 (61.9–87.6)	68.8 (58.7–81.2)	< 0.0001	0
Fibrinogen, g/L	3.3 (2.8–3.9)	3.1 (2.7–3.6)	< 0.0001	64
hs-CRP, mg/L	1.3 (0.7–2.6)	1.3 (0.6–2.5)	0.63	0
ApoB/ApoA1 ratio	0.5 (0.4–0.7)	0.7 (0.6–0.8)	< 0.0001	333
White blood cell count, × 10^9^/L	6.0 (5.1–7.2)	5.4 (4.5–6.4)	< 0.0001	15
Pharmacological treatment				
Renin-angiotensin inhibitors	660 (85.5)	202 (26.2)	< 0.0001	6
Aspirin	749 (97.0)	76 (9.9)	< 0.0001	5
β-Blockers	708 (91.6)	102 (13.2)	< 0.0001	5
Statins	747 (96.9)	127 (16.5)	< 0.0001	9
NSAIDs	11 (1.4)	32 (4.2)	0.001	10
Corticosteroids	23 (3.0)	28 (3.6)	0.48	9
Oral antidiabetic agents	65 (8.4)	47 (6.1)	0.08	6
Insulin treatment	30 (3.9)	24 (3.1)	0.39	10
Glucose tolerance state by OGTT				
Normal glucose tolerance	471 (60.9)	575 (74.6)	< 0.0001	3
New impaired glucose tolerance	153 (19.8)	93 (12.1)	< 0.0001	
New T2DM	72 (9.3)	40 (5.2)	0.002	

Data are presented as n (%) or median (IQR), as appropriate. If not otherwise stated, patient data were retrieved 6–10 weeks after MI

^a^Peripheral artery, rheumatic, pulmonary, and kidney disease were based on self-reported information in standardized questionnaires as well as cancer, whereas the diagnoses of hypertension, diabetes mellitus, and stroke were based on medical history obtained by the study personnel

^b^Defined as a close relative with CVD at < 60 years of age and based on self-reported information in standardized questionnaires

^c^Only assessed in patients without previously known diabetes

*CVD* cardiovascular disease, *FPG* plasma fasting glucose, *2 h-PG* Two hour-postload glucose, *CRP C* reactive protein, *HbA1c* glycohemoglobin A1c, *HDL* high-density lipoprotein, *NSAID* nonsteroidal anti-inflammatory drug, *OGTT* oral glucose tolerance test

Overall, a family history of CVD and smoking were more common in MI patients than among controls, whereas the two groups did not differ by other characteristics, such as history of hypertension, known DM, stroke and depression. In comparison with controls, patients had higher levels of FPG, 2hPG, fibrinogen and white blood cell counts, but there was no significant difference in hs-CRP levels.

Compared to subjects with NGT (patients and controls combined), those with glucose perturbations were older, with lower educational status and a lower proportion of current smokers. Moreover, they had higher BMI and waist circumference as well as significantly higher mannose levels (see Additional file 1: Table S1).

### Mannose levels

In general, median mannose levels increased with tertiles of age (medians: 67.6, 68.1 and 73.0 (μmol/L) in subjects < 50, 50–60 and > 60 years of age, respectively; p = 0.001) and were higher in men than women (median 72.1 vs. 67.1 μmol/L; p = 0.0002).

**Fig. 2:**
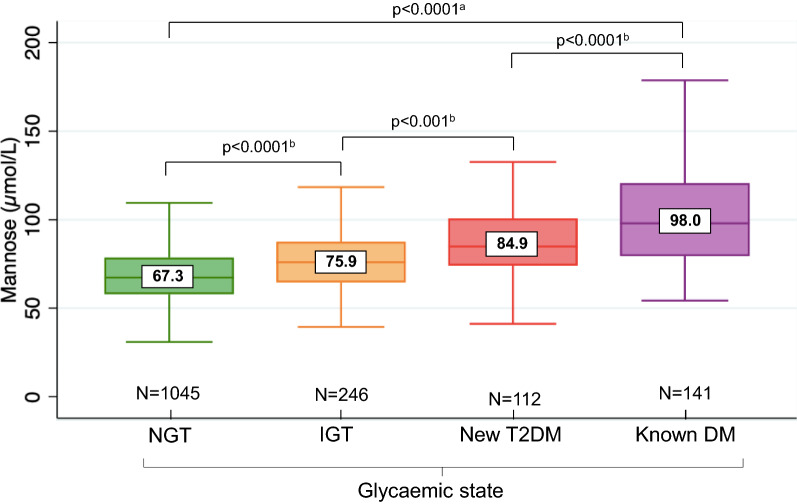
Plasma mannose concentrations across different glycaemic states in the whole population. Box plot shows median (numbers within boxes) and IQR; outliers are not shown. ^a^p-value by Kruskal–Wallis test; ^b^p-value by Mann–Whitney U test. NGT, Normal glucose tolerance; IGT, Impaired glucose tolerance; T2DM, type 2 diabetes mellitus; DM, diabetes mellitus

In the total population (patients and controls) mannose concentrations differed significantly across the four glycaemic groups (NGT, IGT, new T2DM and known DM; p < 0.0001) with a gradual and significant increase in the median levels (NGT = 67.3 μmol/L; IGT = 75.9 μmol/L; new T2DM = 84.9 μmol/L; known DM = 98.0 μmol/L) as shown by pairwise comparisons (Fig. 2).

**Fig. 3:**
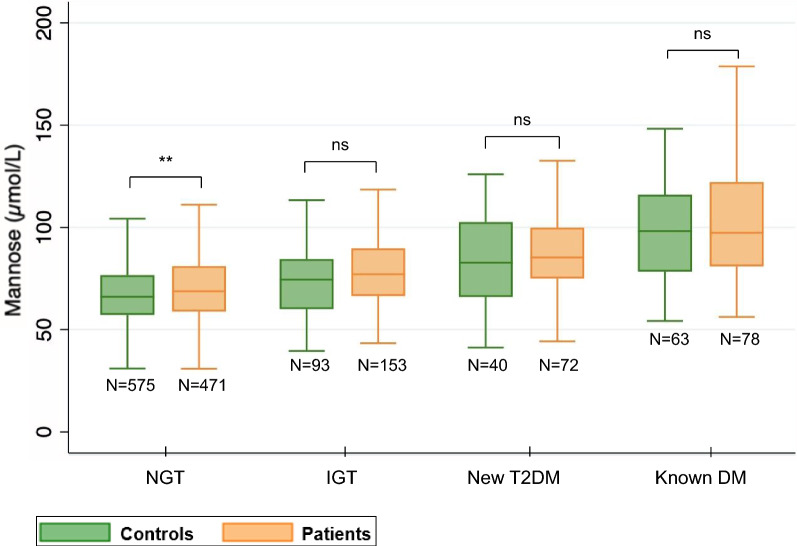
Plasma mannose levels in patients and controls across different glycaemic states. Box plot shows median (line within boxes) and IQR; outliers are not shown. P-values are by Mann–Whitney U test; ^**^p < 0.01; ns, p > 0.05. *NGT* Normal glucose tolerance, *IGT* Impaired glucose tolerance, *T2DM* type 2 diabetes mellitus, *DM* diabetes mellitus

Mannose levels were significantly higher in patients than in controls (median 74.5 *vs* 68.8 μmol/L; p < 0.0001). Within-group differences between patients and controls, even though relatively small, remained significant in NGT (median 68.9 vs 66.1 μmol/L; p = 0.0027) but not in subgroups with glucose perturbations (Fig. 3).

Moreover, both age and sex differences disappeared in the presence of MI or glucose perturbations.

In the total population, mannose levels were significantly correlated (all p < 0.0001) with glycaemic and anthropometric variables (i.e., FPG, 2hPG and HbA1c, fibrinogen, hs-CRP, BMI, waist circumference, and smoking habits), although the correlation coefficients were low. These and related within-group correlations are shown in Table 2.

**Table 2:** Spearman correlation coefficients showing univariate associations of plasma mannose levels with anthropometric and glycaemic parameters in the total population and in patients with and without glucose perturbations

Mannose concentrations			
	Total population (n = 1547)	NGT (n = 1045)	Glucose perturbations (n = 499)
FPG (mmol/L)	0.334	0.223	0.361
2 h-PG (mmol/L)	0.275	0.159	0.164
HbA1c (mmol/mol)	0.283	0.215	0.245
Fibrinogen (g/L)	0.280	0.230	0.345
hs-CRP (mg/dL)	0.280	0.242	0.320
BMI (kg/m2)	0.243	0.224	0.207
Waist circumference (cm)	0.268	0.247	0.215

Groups with glucose perturbations include newly diagnosed impaired glucose tolerance, newly diagnosed type 2 diabetes and known diabetes. All p values are < 0.0001

*NGT* normal glucose tolerance, *FPG* fasting plasma glucose, *2 h-PG* Two hour-postload glucose, *HbA1c* glycohemoglobin A1c, *hs-CRP* high-sensitivity C-reactive protein, *BMI* body mass index

### Association of mannose and a first myocardial infarction

Mannose levels were significantly associated with a first MI (odds ratio, OR: 2.7; 95% confidence interval CI 1.8–4.0), which remained after adjustment (OR: 2.2; 95% CI 1.4–3.5). The estimated OR for one SD increase of mannose levels in patients vs controls was 1.21. This suggests that each SD increase in mannose levels is associated with a 21% higher chance of being in the MI group. A similar association was found within the NGT group (adjusted OR: 2.0; 95% CI 1.2–3.6). There were no significant associations between mannose levels and MI in participants with IGT (adjusted OR: 2.1, 95% CI 0.6–7.2), newly detected T2DM (adjusted OR: 4.1, 95% CI 0.7–24.0) or known DM (adjusted OR: 1.5, 95% CI 0.3–6.5), or when grouping all patients with glucose perturbations together (adjusted OR: 1.8, 95% CI 0.8–3.7).

The optimal cut-off value of mannose concentrations was 71.8 μmol/L, with a sensitivity of 75% and a specificity of 43% (AUC 0.59) (Additional file 2: Fig. S1).

## Discussion

The main findings of the present investigation are that: (1) mannose levels gradually increase with worsening glucose perturbations; (2) mannose is independently associated with a first MI; and (3) this association persists in subjects with NGT, but not in those with glucose perturbations.

Our results support previous works reporting higher mannose levels in individuals with glucose perturbations than in those without [20, 21], further extending them by documenting significantly higher levels in patients with previously known DM compared to those with newly detected T2DM. Even though there is no reference range for mannose concentrations, mainly because of different assay methods, our findings in patients with known DM, 98.0 (79.4−120.7) μmol/L, are comparable to the concentrations measured by assays based on mass spectrometry [18, 20]. Consistent with previous observations [9, 12, 20, 21], we noted a positive linear correlation between mannose and glucose concentrations in the total population. Taken together, this strongly supports the hypothesis that mannose is a strong marker of insulin resistance [11, 12, 22]. Even though insulin resistance was not directly assessed in the current study, it is likely that subjects with increasing levels of glucose intolerance are more insulin resistant than those with NGT and, accordingly, have increasing concentrations of plasma mannose. As recently reported in a cell-specific integrated network analysis [11], the pathophysiological explanation is that, in insulin-resistant patients, dysregulations of enzymes involved in sugar metabolism led to an increase in hepatic glucose utilisation while decreasing mannose phosphorylation. Consequently, mannose accumulates in the cytoplasm and may regurgitate into the blood stream, thus explaining high circulating mannose levels in such patients. This association gains crucial importance when considering that insulin resistance promotes atherogenesis and significantly increases the risk of CVD regardless of the presence of glucose perturbations [13, 23].

The potential role of mannose as a biomarker of CVD has recently gained interest, although it has been investigated only in a few studies so far. A large prospective cohort study reported an association between mannose levels and incident T2DM and CV outcomes including CHD and MI [9]. A validation study, including patients with varying CV risk whose CAD was quantitated by coronary computed angiography, invasive coronary angiography and optical coherence tomography, found an association between plasma mannose and CAD with a vulnerable plaque phenotype, which was independent of traditional CV risk factors [12]. In the effort to minimize the effect of additional confounders the present study selected patients with a first MI, i.e. with a rather benign CV risk profile compared with matched controls. Our results support the hypothesis that mannose is a novel marker of clinically manifest coronary atherosclerosis, on the basis of pathophysiological mechanisms that do not depend on acute settings. In this regard, in the present population hs-CRP, which is a marker of acute inflammation, was not significantly correlated with a first MI.

Interestingly, the association between mannose levels and a first MI was no longer significant in the subgroup of subjects with altered glycaemic state, although its magnitude was similar to that found in patients with NGT (OR 1.8 vs 2.0). A reasonable pathophysiological interpretation of these findings may be that, in subjects with glucose perturbations, the association of mannose with CAD is, at least partially, trumped by the presence of severe insulin resistance, which is its main regulator [10]. The absence of this association in the group with glucose perturbations could also be explained by the fact that mannose might mediate the CV risk in these patients. It should be acknowledged that the number of patients in each group with glucose perturbations is smaller than in the NGT group, introducing the possibility of an excessive sample size imbalance; however, this is unlikely, since the association remains non-significant even when grouping all patients with glucose perturbations together. Further, one could speculate whether the association is attenuated also by grouping patients with newly detected glucose perturbations with those with known diabetes, as the latter might be treated with drugs affecting mannose levels and/or insulin sensitivity. Thus, from a clinical point of view, the assay of mannose among subjects without apparent glucose perturbations may represent a valuable tool for the early detection of patients at high risk for coronary events and possibly encourage more aggressive prevention strategies towards selected groups of patients.

### Strengths and limitations

To the best of our knowledge, this is the only investigation addressing the association between plasma mannose levels in a large group of subjects with different and well-characterized degrees of glucose tolerance. The major strength of the present investigation is its large, well phenotyped, and homogeneous population [14]. The glycaemic state of all participants without established DM was carefully investigated by the means of a standard OGTT, which allowed us to detect a significant proportion of subjects with previously undetected glucose perturbations (i.e., new IGT or new T2DM) among both patients and controls. IGT is a state of intermediate glucose intolerance that can only be diagnosed with an OGTT and that has a high prognostic value for future CV events in patients with CAD [6, 24–29]. The use of HPLC–MS-MS for the assay of plasma mannose not only eliminates the interference of blood glucose, which is present at much higher concentrations, but thanks to its adequate selectivity, reproducibility (relative SD < 10%), accuracy (96–104%), and limited cost, represents a suitable tool for mannose quantification in clinical settings [12, 18].

Some limitations should be considered. First, the proportion of subjects with glucose perturbations is somewhat lower compared with previous works, reporting approximately two thirds of coronary patients being affected by either IGT or T2DM [30, 31]. This discrepancy is likely explained by the selection of a healthier and younger population in the present study, which also contributes to the imbalance in size between the subgroups of subjects with and without glucose perturbations [17]. The large sample size and the consistency of results when grouping all patients with glucose perturbations together do, however, suggest that the investigation is adequately powered. Moreover, there is a lack of information on the impact that may be exerted by pharmacological treatments, such as glucose-lowering drugs prescribed in patients with known DM, on plasma mannose concentrations. However, the proportion of patients with known DM is low and comparable between patients and controls (10.1% vs. 8.2%).

Finally, because of the observational design of the PAROKRANK study [14], the current investigation cannot demonstrate the presence of a causal relationship between high mannose and MI. Hence, further studies are needed to confirm the importance of measuring mannose in a clinical setting, for instance by evaluating its impact on future CV events and mortality in subjects with different glycaemic states.

## Conclusions

In conclusion, the current data support the hypothesis that high mannose might represent a novel, independent and possibly more sensitive marker of risk for MI than glucose-related expressions for glucose perturbations. Therefore, the assessment of mannose concentrations could represent a valuable clinical tool for improving CV risk stratification and allow early personalized intervention in high-risk patients.

## Supplementary Information

**Additional file 1:** **Table S1**. Baseline characteristics of patients with and without dysglycaemia.

**Additional file 2:** **Figure S1**. Adjusted receiver operator characteristics (ROC) curve testing the diagnostic performance of plasma mannose concentrations in diagnosing a first MI. AUC (Area under the curve): 0.59. Optimal cut-off value: 71.8 μmol/L, with a sensitivity of 75% and a specificity of 43%.

## Data Availability

The data underlying this study are available from GF and LR upon reasonable request.

## Abbreviations

2 h-PG: Two hours post-load glucose
AUC: Area under the curve
CAD: Coronary artery disease
CV: Cardiovascular
CVD: Cardiovascular disease
DM: Diabetes mellitus
FPG: Fasting plasma glucose
HPLC–MS-MS: High-performance liquid chromatography coupled to tandem mass spectrometry
HbA1c: Glycated haemoglobin A1c
hs-CRP: High-sensitivity C-reactive protein
IGT: Impaired glucose tolerance
MI: Myocardial infarction
NGT: Normal glucose tolerance
OGTT: Oral glucose tolerance test
OR: Odds ratio
P: Plasma
PAROKRANK: Periodontitis and Its Relation to Coronary Artery Disease
ROC: Receiver operator characteristics
T2DM: Type 2 diabetes mellitus
WHO: World Health Organization
