# Supplementary material for: Plasma mannose as a novel marker of myocardial infarction across different glycaemic states: a case control study
Source: Cardiovasc Diabetol. 2022 Sep 23;21:195. doi: 10.1186/s12933-022-01630-5 (PMC9508730; doi:10.1186/s12933-022-01630-5)
Supplement: Supplementary file 1 — Additional file 1: Table S1. Baseline characteristics of patients with and without dysglycaemia. [file 12933_2022_1630_MOESM1_ESM.docx]

**Table S1** Baseline characteristics of patients with and without dysglycaemia

|  | No dysglycaemia (n=1045) | Dysglycaemia (n=499) | p-value |
| --- | --- | --- | --- |
| Age, years | 63 (56 – 67) | 65 (60 – 69) | < 0.001 |
| Male sex | 846 (81.0) | 404 (81.0) | 1.00 |
| Education level |  |  |  |
| 1-12 years | 639 (61.4) | 341 (68.6) | 0.006 |
| University | 402 (38.6) | 156 (31.4) |  |
| Known family history of CVD^a^ | 326 (31.2) | 145 (29.1) | 0.39 |
| Smoking habits (patients at admission) |  |  |  |
| Current | 440 (42.5) | 166 (33.5) |  |
| Previous (>1 month) | 483 (46.6) | 286 (57.7) | 0.05 |
| Never | 113 (10.9) | 44 (8.9) |  |
| Waist circumference. cm | 97.0 (14.0) | 102.0 (14.8) | < 0.001 |
| Body Mass Index, kg/m^2^ | 26.2 (24.1 – 28.4) | 27.5 (24.7 – 30.6) | < 0.001 |
| Laboratory values |  |  |  |
| Triglycerides, mmol/L | 1.1 (0.8-1.5) | 1.2 (0.9-1.8) | < 0.001 |
| HDL-cholesterol, mmol/L | 1.3 (1.1 – 1.6) | 1.2 (1.0 – 1.5) | < 0.001 |
| FPG, mmol/L | 5.4 (5.0 – 5.8) | 6.4 (5.7 – 7.3) | < 0.001 |
| Mannose, μmol/L | 67.3 (57.9 – 78.6) | 80.9 (69.4 – 98.4) | < 0.001 |
| Fibrinogen, g/L | 3.1 (2.7 – 3.6) | 3.3 (2.9 – 3.9) | < 0.001 |
| hs-CRP, mg/L | 1.2 (0.6 – 2.3) | 1.6 (0.8 – 3.3) | < 0.001 |

Data are presented as n (%) or median (IQR). If not otherwise stated, patient data were retrieved 6-10 weeks after MI. Dysglycaemia group includes newly diagnosed impaired glucose tolerance, newly diagnosed type 2 diabetes and known diabetes.

^a^ Defined as a close relative with CVD at <60 years of age and based on self-reported information in standardized questionnaires.

CVD, cardiovascular disease; HDL, high-density lipoprotein; FPG, fasting plasma glucose; hs-CRP, high-sensitivity C-reactive protein.
