# Supplementary material for: Plasma mannose as a novel marker of myocardial infarction across different glycaemic states: a case control study
Source: Cardiovasc Diabetol. 2022 Sep 23;21:195. doi: 10.1186/s12933-022-01630-5 (PMC9508730; doi:10.1186/s12933-022-01630-5)
Supplement: Supplementary file 2 — Additional file 2: Figure S1. Adjusted receiver operator characteristics (ROC) curve testing the diagnostic performance of plasma mannose concentrations in diagnosing a first MI. AUC (Area under the curve): 0.59. Optimal cut-off value: 71.8 μmol/L, with a sensitivity of 75% and a specificity of 43%. [file 12933_2022_1630_MOESM2_ESM.pdf]

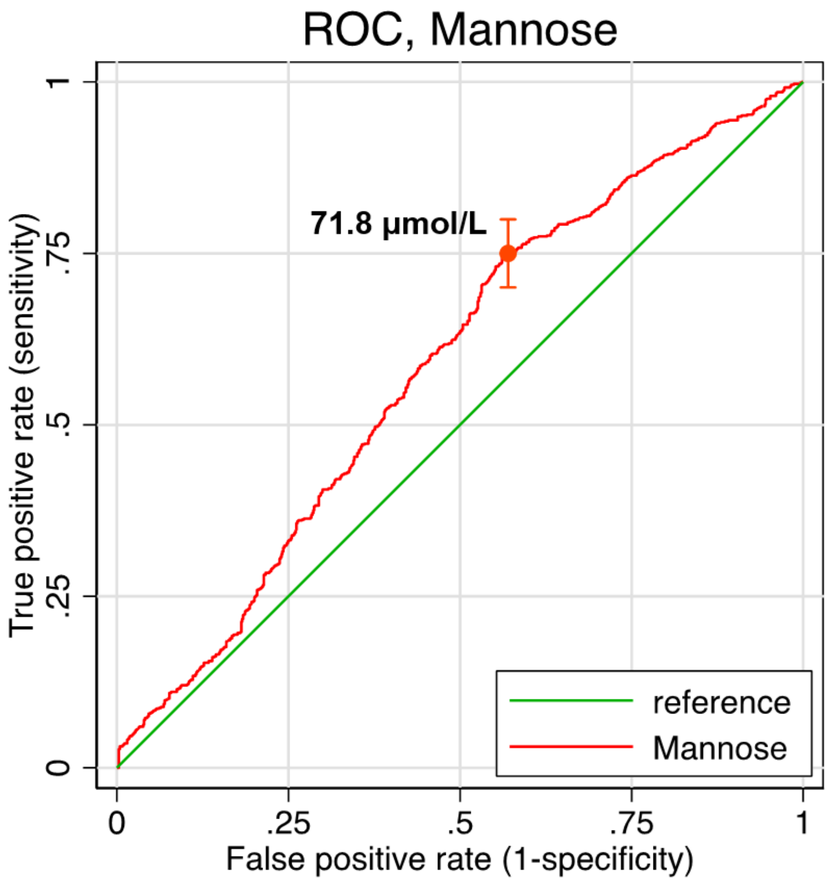

Additional file 2: Figure S1. Adjusted receiver operator characteristics (ROC) curve testing the diagnostic performance of plasma mannose concentrations in diagnosing a first MI. AUC (Area under the curve): 0.59. Optimal cut-off value: 71.8 µmol/L, with a sensitivity of 75% and a specificity of 43%.
